# A Non-Contact Privacy Protection Bed Angle Estimation Method Based on LiDAR

**DOI:** 10.3390/s25072226

**Published:** 2025-04-02

**Authors:** Yezhao Ju, Yuanji Li, Haiyang Zhang, Le Xin, Changming Zhao, Ziyi Xu

**Affiliations:** 1 School of Optoelectronics, Beijing Institute of Technology, Beijing 100086, China; 2 Nanjing Research Institute of Electronics Technology, Nanjing 210039, China

**Keywords:** bed angle detection, LiDAR, point cloud, A2J-Posenet

## Abstract

Accurate bed angle monitoring is crucial in healthcare settings, particularly in Intensive Care Units (ICUs), where improper bed positioning can lead to severe complications such as ventilator-associated pneumonia. Traditional camera-based solutions, while effective, often raise significant privacy concerns. This study proposes a non-intrusive bed angle detection system based on LiDAR technology, utilizing the Intel RealSense L515 sensor. By leveraging time-of-flight principles, the system enables real-time, privacy-preserving monitoring of head-of-bed elevation angles without direct visual surveillance. Our methodology integrates advanced techniques, including coordinate system transformation, plane fitting, and a deep learning framework combining YOLO-X with an enhanced A2J algorithm. Customized loss functions further improve angle estimation accuracy. Experimental results in ICU environments demonstrate the system’s effectiveness, with an average angle detection error of less than 3 degrees.

## 1. Introduction

Patients spend a significant portion of their time in hospital beds, making the study of bed status essential for understanding how it affects health outcomes and recovery rates [1]. Research indicates a strong correlation between the head-of-bed elevation angle and the incidence of hospital-acquired infections (HAIs) [2], such as ventilator-associated pneumonia (VAP). The inhalation of oropharyngeal secretions is a critical factor in the development of VAP, with up to 45% of healthy individuals potentially aspirating these secretions during sleep. The “Guidelines for the Diagnosis and Treatment of Hospital-Acquired Pneumonia and Ventilator-Associated Pneumonia in Chinese Adults” (2018) recommend elevating the bed head to an angle of 30° to 45° for mechanically ventilated patients in the Intensive Care Unit (ICU) without contraindications, as this can effectively reduce gastric reflux and aspiration, serving as a crucial preventative measure against adult VAP [3].

The objective of our research is to support nursing staff by providing a non-invasive means of monitoring bed elevation angles, thereby helping to prevent pneumonia and other complications without increasing the risk of additional HAIs [4,5]. Existing methods for bed angle monitoring also face several limitations. For instance, internal bed sensors, such as pressure-sensitive bedsheets [6], are not universally available in all hospital beds and may lack the accuracy required for precise angle detection in dynamic settings. Additionally, modified compasses or lead hammers based on gravity and geometric principles have been proposed for bed angle monitoring. However, these devices rely on manual operation, which is not only time-consuming but also impractical for continuous monitoring in busy hospital environments. Moreover, vibrations or movements in the hospital environment can significantly affect the accuracy of these mechanical devices, resulting in unreliable angle measurements [7]. Camera-based edge detection methods offer a viable solution for detecting multiple beds efficiently; however, they pose significant privacy risks and ethical concerns, making them difficult to implement in clinical settings [6].

To address these issues, Yun Li et al. proposed an edge detection method using depth images collected by Kinect 360 to monitor bed states [8]. However, this approach is unreliable when the bed edges are obscured by patients or bedding. Katayama, H. et al. introduced a plane fitting method using Kinect V2 depth images to detect the head-of-bed elevation angle [9], but this technique is limited in environments where the bed surface is not consistently planar due to the presence of patients and bedding [10].

In addition to these technical challenges, there is also a need for continuous, real-time monitoring solutions that do not compromise patient privacy. Traditional camera-based systems often raise ethical concerns related to patient dignity and confidentiality. Furthermore, existing solutions may fail to provide accurate measurements under dynamic conditions, such as when patients move frequently or when bedding is adjusted.

To address these challenges, our study proposes a non-contact, privacy-preserving bed angle detection system based on LiDAR technology. Unlike camera-based systems, our approach avoids visual surveillance, thereby respecting patient privacy. Moreover, compared to internal sensors and other contact-based methods, our system is non-invasive, cost-effective, and scalable for monitoring multiple beds simultaneously. By leveraging advanced techniques such as coordinate system transformation, plane fitting, and deep learning, our method provides accurate and real-time bed angle monitoring, making it a practical solution for modern healthcare environments.

In this paper, we present a novel approach using an Intel L515 laser radar to collect three-dimensional point cloud data and implement a top-down bed angle detection algorithm based on YOLO-X and an improved A2J. Using these three-dimensional point cloud data, we estimate key bed points, the head-of-bed elevation angle, and bed height. The Intel L515 device was installed in the ward, allowing real-time remote monitoring of the bed state via a graphical user interface (GUI). This system not only addresses the limitations of previous methods but also ensures high accuracy and reliability even under complex environmental conditions. Moreover, our approach respects patient privacy by avoiding direct visual surveillance, thus offering a more ethical and practical solution for continuous bed angle monitoring in clinical settings.

## 2. Materials and Methods

### 2.1. Point Cloud Scene Acquisition and Data Preprocessing

The Intel RealSense L515 (Intel Corporation, Santa Clara, CA, USA)represents a significant advancement in depth sensing technology, leveraging time-of-flight (ToF) principles to deliver precise distance measurements for objects within its field of view. By calculating the time it takes for emitted laser signals to return after reflecting off surfaces, this sensor generates detailed 3D maps of its environment. This method is especially advantageous due to its high integration capabilities, swift response times, and minimal environmental impact, making it ideal for use in sensitive indoor medical environments where accuracy and reliability are paramount.

What sets the L515 apart from other ToF depth sensors is its innovative Micro-Electro-Mechanical System (MEMS) mirror scanning technology, which contributes to its compact size and makes it the world’s smallest high-resolution imaging LiDAR device. This technology not only ensures portability but also enhances efficiency. The L515 achieves higher laser power efficiency compared to its counterparts while consuming less than 3.5 watts during operation. It offers impressive specifications, including a depth resolution of 1024 × 768 pixels, an operational range extending from 0.25 m to 9 m, and support for frame rates up to 30 frames per second, providing clear and detailed point cloud data.

**Figure 1:**
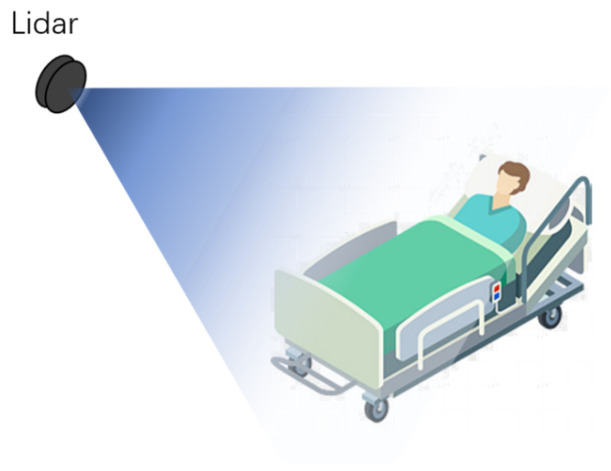
Laser radar deployment diagram.

**Figure 2:**
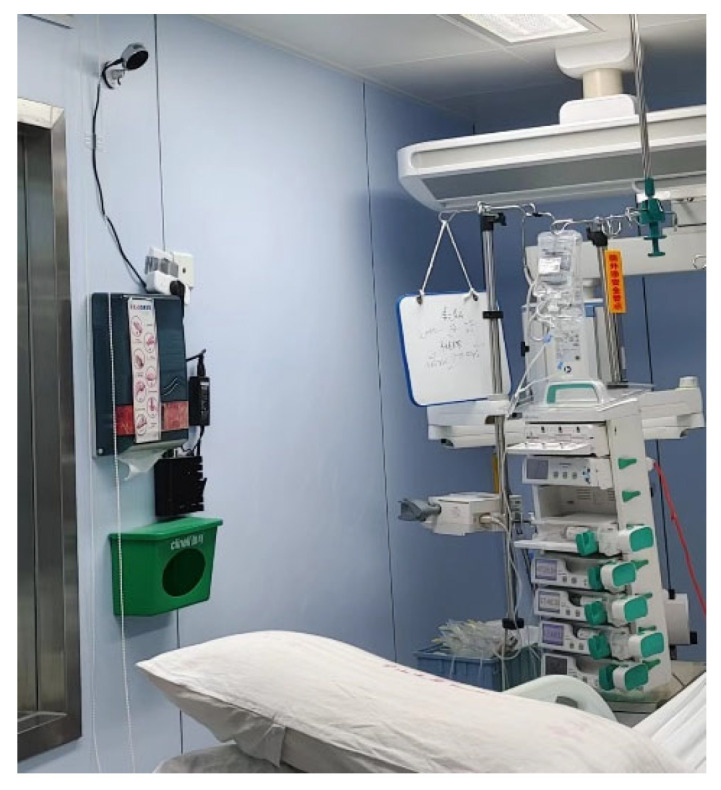
Laser radar deployment in ICU.

To maximize the effectiveness of the L515 in medical settings, careful consideration must be given to its placement. Given the dynamic nature of healthcare environments, characterized by frequent movement and the presence of various medical apparatus, strategic positioning is crucial. The sensor is ideally mounted on a wall at a height of approximately two meters above the ground, angled downwards. This setup minimizes occlusions and obstructions, ensuring optimal visibility and accuracy in capturing bed elevation angles and overall room layout. Figure 1 and Figure 2 illustrate this configuration, highlighting how such positioning facilitates seamless data acquisition and preprocessing for applications in medical care.

### 2.2. Transformation of Camera Coordinate System to World Coordinate System

In this paper, the transformation from the camera coordinate system to the world coordinate system is realized by calculating the transformation matrix. Random sample consensus [11] is used to fit the ground of the shooting scene. The plane equation of the ground can be expressed as in Equation (1):

(1) ax+by+cz+d=0

The unit normal vector of the ground in the lidar coordinate system is expressed as follows:

(2) $${\vec{r}}_{lidar}=\frac{\left(a,b,c\right)}{\sqrt{a^{2}+b^{2}+c^{2}}}$$

The normal vector of the ground in the world coordinate system is ${\vec{r}}_{ground}=\left(0,0,1\right)$, and $\vec{r}={\vec{r}}_{lidar}\times {\vec{r}}_{ground}$. The transformation matrix *R* is expressed as follows:

(3) $$R=I\cdot \cos\theta +\left(1-\cos\theta \right)\cdot \vec{r}\cdot {\vec{r}}^{T}+\sin\theta \cdot \left(\begin{array}{ccc} 0 & -{\vec{r}}_{z} & {\vec{r}}_{y}\\ \;{\vec{r}}_{z} & 0 & -{\vec{r}}_{x}\\ -{\vec{r}}_{y} & \;{\vec{r}}_{z} & 0 \end{array}\right)$$

where I is the unit vector, and *θ* is the angle between ${\vec{r}}_{lidar}$ and ${\vec{r}}_{ground}$. The key points of the human body output by the three-dimensional pose estimation module are transformed into the representation of the world coordinate system through matrix transformation, which is more convenient for feature extraction [12].

**Figure 3:**
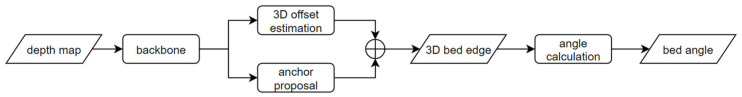
Bed angle detection network frame diagram.

The method comprises three main components: a bed target detection module, a key point estimation module, and a bed angle calculation module. Figure 3 illustrate the overall network framework, with the network input being a depth image of 288 × 288 pixels. The three-dimensional pose estimation module includes a backbone network and two branches, utilizing the concept of anchor-weighted voting to locate the key points of the bed angle. The network mainly consists of three parts, the backbone network Resnet50 and two prediction branches, where the two prediction branches include offset prediction branch and anchor weight prediction branch. A human depth map is input, and its corresponding 2D anchor points are generated first. Then, the depth map is input into the backbone network to extract features, the features are input into two prediction branches to obtain the network’s output, and finally the anchor point coordinates are aggregated with the output of each branch in the post-processing module to obtain the predicted key point coordinates.

**Figure 4:**
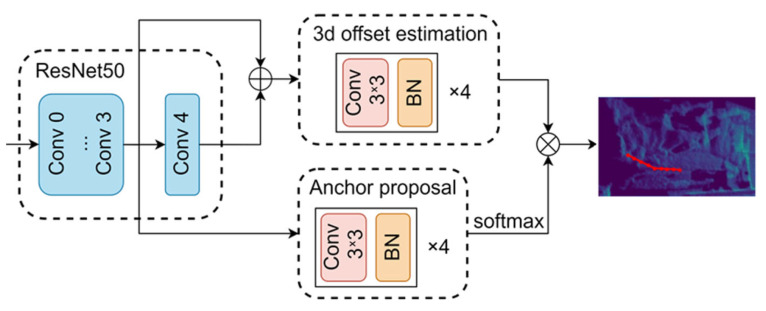
The framework of the key point estimate network.

When the head of the bed is elevated, an angle is formed between the head and the foot of the bed. Nine key points along the bed edge are detected: the first four key points represent the head of the bed, the next four key points represent the foot of the bed, and the central key point represents the corner of the bed. The angle measurement is derived through vector operations and statistical analysis. The bed angle calculation module performs geometric calculations based on the three-dimensional bed edge, ultimately generating the head-of-bed elevation angle. In Figure 4, the red dots represent the key points estimated by the Bed Angle Detection Network. By connecting these points and computing their angular mean, the final head-of-bed angle is derived.

The region enclosed by the bounding box is cropped and padded into an image with a 1:1 aspect ratio with zero-pixel padding, ensuring that the bed retains its original proportions and preventing deformation when the image is reshaped within the network. This enhances the network’s robustness to varying bed states.

**Figure 5:**
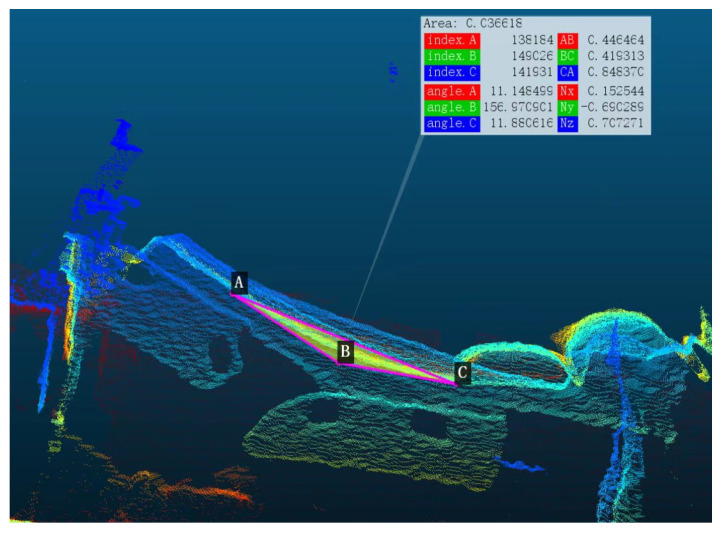
The key point diagram of the bed edge.

For the annotation of bed angle key points (as shown in Figure 5), we used CloudCompare V2.13 alpha software as the annotation tool. Specifically, we utilized its angle measurement tool to accurately measure the bed angle at point B, ensuring that the annotated angle matched the actual test values with an error of less than 1°. The angle measurement tool in CloudCompare allowed us to select three points (A, B, and C) corresponding to the head, corner, and tail of the bed, respectively. These points were recorded as the original annotations. Based on the positions of points A, B, and C, the remaining six key points were filled in through linear relationships, thereby improving the accuracy of bed angle prediction. This approach ensured that the annotated key points were consistent with the geometric structure of the bed, enhancing the reliability of the dataset for training and evaluation.

This paper introduces two loss functions based on the positioning of anchor points and key points. The loss function around the information anchor is designed to locate the anchor point relative to the key point. The weight of the anchor point and the coordinate offset between the key point and the anchor point are then used to compute the loss of the key point. The expression for the loss around the information anchor is as follows:

(4) $$\begin{array}{cc} {loss}_{wkp} & =\sum_{j\in J}L_{\tau }\sum_{a\in A}\left(\overset{\sim_{j}\left(a\right)S\left(a\right)}{P}-T_{j}\right) \end{array}$$

In the formula, $\overset{\sim_{j}\left(a\right)}{P}$ is the weight from anchor point a to key point *j*, $S\left(a\right)$ is the coordinate of anchor point a, $T_{j}$ is the actual position of key point *j*, and $L_{\tau }\left(x\right)$ is the SmoothL1-like loss function. It is given by the following formula:

(5) $$L_{\tau }\left(x\right)=\left\{\begin{array}{cc} \frac{1}{2\tau }x^{2}, & \left|x\right|<\tau \\ \left|x\right|-\frac{\tau }{2}, & \left|x\right|>\tau \end{array}\right.$$

where *τ* is 1. When calculating the positioning loss of key points, the depth value is extended to 50 times the actual depth value (m), so that the plane coordinate and the depth coordinate are in the same order of magnitude. The improved key point positioning loss is as follows:

(6) $$\begin{array}{cc} {loss}_{anchor} & =\sum_{j\in J}L_{\tau }\sum_{a\in A}\left(\overset{\sim_{j}\left(a\right)\left(S\left(a\right)+O_{j}\left(a\right)\right)}{P}-T_{j}\right) \end{array}$$

$O_{j}\left(a\right)$ denotes the coordinate deviation from anchor point a to key point *j*.

In order to further constrain the position of the key points, a linear loss function is introduced to optimize the straightness error of the two straight lines at the head and tail of the bed formed by the key points.

(7) $$loss_{line}=6-\sum_{i=1}^{3}Sim\left(A_{head},A_{4i}\right)-\sum_{i=5}^{7}Sim\left(A_{end},A_{4i}\right)$$

Vector *A* represents the vector composed of the key points of the bed edge obtained by the key point network inference. $Sim\left(A_{head},A_{4i}\right)$ represents the cosine similarity between the bed head vector $A_{head}$ and the bed edge key point vector $A_{4i}$, and $Sim\left(A_{end},A_{4i}\right)$ represents the cosine similarity between the bed tail vector $A_{end}$ and the bed edge key point vector $A_{4i}$ obtained by network reasoning.

(8) $$A_{head}=\sum_{i=1}^{3}A_{4i}$$

(9) $$A_{end}=\sum_{i=5}^{7}A_{4i}$$

The bed edge key point vector $A_{4i}$ is a directed line segment from the predicted bed edge key point 4 to *i*.

The cosine similarity is defined as follows:

(10) $$Sim\left(A,B\right)=\frac{AB}{\left|\left|A\right|\right|\times \left|\left|B\right|\right|}=\frac{\sum_{i=1}^{n}\left(A_{i}\times B_{i}\right)}{\sqrt{\sum_{i=1}^{n}A_{i}^{2}}\times \sqrt{\sum_{i=1}^{n}B_{i}^{2}}}$$

Finally, in order to further optimize the key point position, an angle loss function is introduced to optimize the angle error.

(11) $$loss_{angle}=\beta -\alpha$$

(12) $$\alpha =\arccos\frac{A_{head}A_{end}}{\left|\left|A_{head}\right|\right|\times \left|\left|A_{end}\right|\right|}$$

(13) $$\beta =\arccos\frac{B_{head}B_{end}}{\left|\left|B_{head}\right|\right|\times \left|\left|B_{end}\right|\right|}$$

In the formula, vector *A* represents the vector composed of the key points of the bed edge obtained by network prediction, and vector *B* represents the vector composed of the key points of the actual bed edge obtained by labeling.

(14) $$B_{head}=\sum_{i=1}^{3}B_{4i}$$

(15) $$B_{end}=\sum_{i=5}^{7}B_{4i}$$

The total loss of the bed edge key point estimation network is expressed as follows:

(16) $$loss=loss_{wkp}+loss_{anchor}+\lambda_{1}loss_{line}+\lambda_{2}loss_{angle}$$

In order to balance the four losses, two balance factors $\lambda_{1}=1000$ and $\lambda_{2}=10$ are introduced.

**Figure 6:**
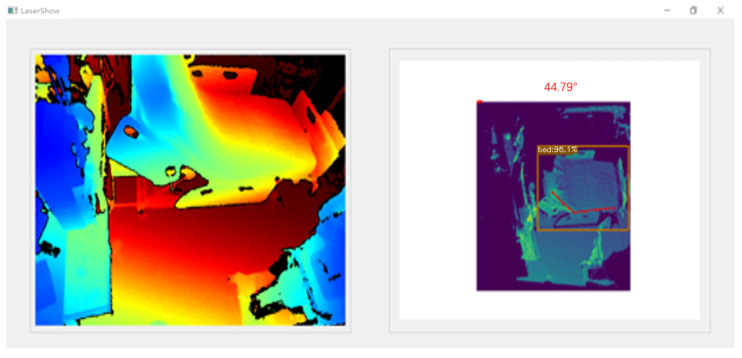
Bed angle detection interface.

After estimating the key points of the nine bed edges, we calculate the average angle between these points and take the mean over twenty consecutive frames to obtain the final angle result. The bed angle detection interface is illustrated in Figure 6, which presents a comprehensive visualization of the detection process. On the left side of the interface, the raw LiDAR point cloud data is displayed with pseudo-coloring based on the distance from the LiDAR sensor, providing an intuitive representation of the spatial distribution of the scanned environment. The right side shows the algorithm-processed results, where the point cloud data is mapped onto a 2D depth plane with pseudo-coloring for enhanced visualization clarity. This processed view clearly highlights the detected bed position, along with the calculated bed angle overlaid on the depth image, enabling real-time monitoring and verification of the angle measurement. The side-by-side comparison in Figure 6 effectively demonstrates the transformation from raw sensor data to actionable clinical information, showcasing the system’s ability to accurately localize the bed and compute its elevation angle in a visually interpretable manner.

## 3. Results

### 3.1. The Experiment Setup

The experimental system operates on a Windows 10 64-bit platform, providing stability and performance for complex computational tasks. The hardware includes an Intel Core i7-10700 CPU and an NVIDIA GeForce RTX 3080 Ti GPU, ensuring efficient execution of real-time deep learning applications. For software, we used PyTorch 1.10, complemented by CUDA 11.3.1 and cuDNN 8.2.1 to optimize GPU acceleration. Input images were standardized to 288 × 288 pixels to ensure dataset consistency and facilitate effective model training.

The Intel RealSense L515 LiDAR sensor was mounted on a wall at a height of 2 m, angled downward at 30 degrees to the horizontal plane. This configuration minimizes occlusions and ensures optimal coverage of the bed area, even in dynamic healthcare environments with frequent movement of medical staff and equipment. Preliminary tests confirmed that the sensor’s field of view (FOV) fully covered the bed area, and adjustments were made to avoid interference from reflective surfaces.

In the laboratory, we simulated various bed configurations, including different elevation angles and occlusion scenarios, to collect a diverse dataset. In the ICU, data were collected from three beds over six months, capturing real-world conditions such as empty beds, occupied beds, and dynamic scenarios with moving medical equipment. The collected point cloud data were preprocessed to remove noise and outliers, and aligned with the world coordinate system using RANSAC plane fitting. This ensured accurate representation of the bed surface in the 3D point cloud, enabling precise angle estimation.

### 3.2. The Training Process

Training was performed through fine-tuning over a total of 300 epochs. An initial phase of 10 epochs was dedicated to warming up the learning rate, a common practice in deep learning to stabilize the training process. During this warm-up phase, the learning rate is gradually increased from a small initial value, allowing the model to adapt to the data distribution without being overwhelmed by large gradient updates. This approach helps prevent early overfitting and ensures smoother convergence during the main training phase.

Over the subsequent 290 epochs, the Adam optimizer dynamically adjusted the learning rate, optimizing the model’s parameters for better performance. The learning rate warm-up strategy, combined with Adam’s adaptive learning rate mechanism, effectively balanced the trade-off between exploration and exploitation in the parameter space. Throughout this process, the best-performing model was saved based on validation accuracy, ensuring that the final model was the most effective iteration. In addition to simulating a ward environment within the laboratory to collect point cloud scene data, we also gathered real-world data from three Intensive Care Unit (ICU) wards at the First Affiliated Hospital of Sun Yat-sen University. Specifically, data were collected from ICU beds numbered 15, 16, and 17, covering various angles, baffle positions, and scenarios ranging from empty to occupied beds. This comprehensive approach ensured a diverse and representative dataset, capturing the complexities and variabilities present in actual medical settings.

### 3.3. The Training Result

After thorough labeling and extensive data augmentation techniques to enhance the dataset’s richness and diversity, a total of 3552 samples were compiled into a hospital bed angle dataset. To ensure the robustness and generalizability of the model, 80% of these samples were allocated for training purposes, while the remaining 20% were reserved for testing. This meticulous preparation of the dataset and rigorous training regimen laid a solid foundation for developing an accurate and reliable model capable of determining bed elevation angles in hospital environments.

**Figure 7:**
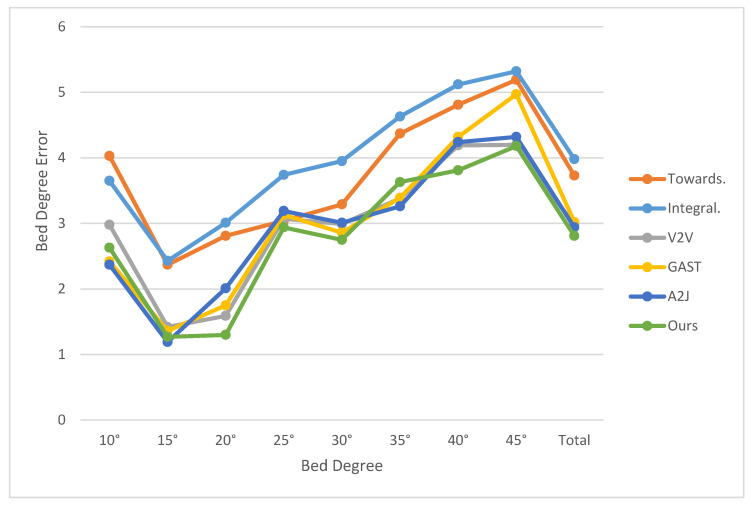
Comparison of results of different methods on bed angle dataset.

In the experimental phase, we set the bed angles in increments of 5 degrees, ranging from 10 to 45 degrees, establishing a total of nine experimental groups for the head-of-bed elevation angles. This systematic approach ensured a comprehensive range of scenarios, covering typical bed configurations encountered in hospital settings. In the laboratory environment, the angle measured by a high-precision angle measuring instrument served as the true value for the bed angle. Similarly, in the ICU ward environment, the elevation angle displayed by the electric nursing bed was used as the true value. The test results, computed using our bed angle measurement algorithm, are presented in Figure 7.

The experimental results demonstrate the effectiveness of our LiDAR-based bed angle detection system across a range of elevation angles from 10° to 45°. As shown in Table 1, our method achieves an average angle detection error of 2.81°, outperforming several existing approaches, including Towards (3.73°), Integral (3.98°), V2V (2.97°), GAST (3.02°), and A2J (2.94°). Notably, our system exhibits superior performance at lower angles (e.g., 1.27° at 15° and 1.30° at 20°), which are critical for preventing complications such as ventilator-associated pneumonia (VAP) and pressure ulcers. Visible light camera-based methods rely on 2D image analysis, which is highly susceptible to occlusions and lighting variations. In contrast, our LiDAR-based system uses 3D point cloud data, which is less affected by occlusions and provides more robust angle estimation, even in dynamic environments. Traditional mechanical measurement methods, such as inclinometers or protractors, require direct contact with the bed and are often limited to single-bed scenarios. These methods are impractical for continuous monitoring in hospital settings.

**Figure 8:**
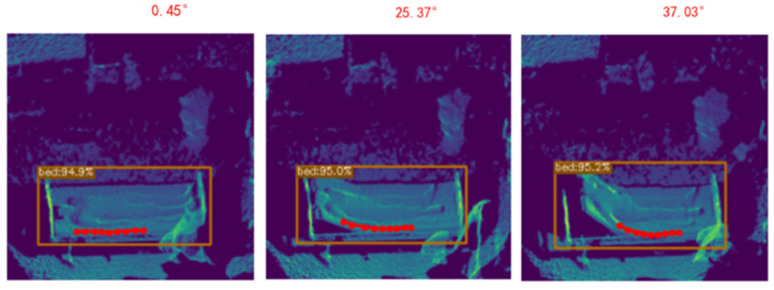
Real-time bed angle predict result in lab.

**Figure 9:**
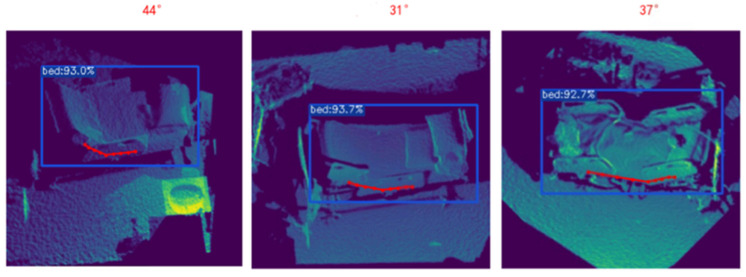
Real-time bed angle predict result in ICU.

The experimental results are presented in Figure 8 and Figure 9, which visually illustrates the performance of our algorithm across all tested angles. The red dots represent the key points estimated by the Bed Angle Detection Network. By connecting these points and computing their angular mean, the final head-of-bed angle is derived. This chart includes detailed comparisons between the estimated and true values, highlighting the accuracy and reliability of our system. Furthermore, it provides insights into any potential sources of error or areas for improvement, guiding future refinements of the algorithm. Through this structured and thorough experimental design, we aimed to develop a robust and dependable solution for accurately measuring bed elevation angles in various medical environments.

We have successfully deployed our advanced bed angle measurement system for long-term monitoring in an Intensive Care Unit (ICU) ward. This deployment marks a significant step towards enhancing patient care by ensuring that bed angles are adjusted accurately and promptly according to clinical requirements. The system has been operational for six months, continuously recording daily changes in bed angles and monitoring whether adjustments are made in a timely and appropriate manner. As shown in Table 2, the system demonstrates reliable performance across different bed angles, with measured results closely matching the target angles. When the bed barrier is in the up position, the average error remains within 1.1° to 3.5°, while with the barrier down, errors range from 0.8° to 4.8°, indicating consistent accuracy under varying conditions. This extended period of operation has allowed us to gather extensive data on bed angle variations and their correlation with patient care routines. The continuous monitoring ensures that healthcare providers can maintain optimal bed configurations, which is crucial for preventing complications such as pressure ulcers and respiratory issues.

**Figure 10:**
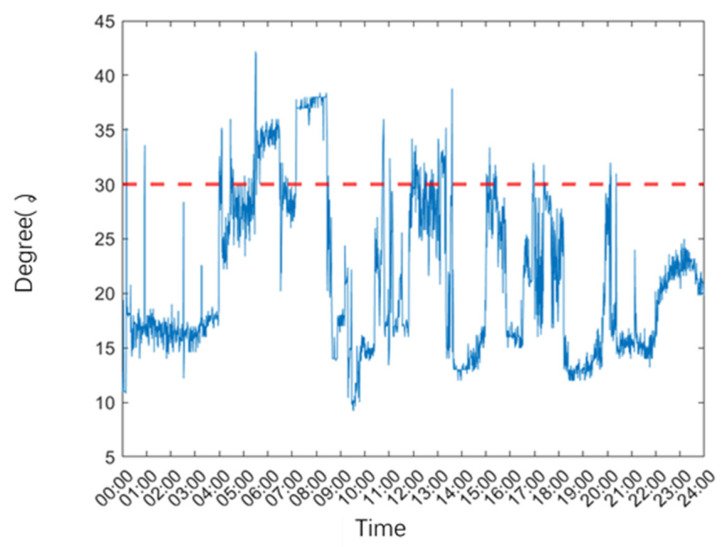
One day bed angle changes in ICU.

This detailed record illustrates the system’s capability to capture and report bed angle changes with high precision. By providing real-time data and historical trends, the system enhances the overall quality of care by ensuring that bed angles are consistently managed in accordance with best practices. Figure 10 visually demonstrates the system’s reliability in tracking bed angle variations, particularly highlighting how the 30-degree elevation serves as a critical clinical threshold. Maintaining this angle is essential for optimizing patient outcomes, as it effectively balances benefits such as reducing ventilator-associated pneumonia risk, improving respiratory function, and minimizing pressure ulcer formation while avoiding excessive elevation that could lead to patient discomfort or sliding. The system’s continuous monitoring ensures adherence to this optimal angle, reinforcing its clinical value. 

## 4. Discussion

In this study, we introduced an innovative bed angle detection algorithm specifically designed for medical settings, leveraging advanced 3D point cloud data captured by laser radar technology. This approach addresses the critical need for precise and reliable bed angle measurements in hospital environments, where accurate patient positioning is essential for effective care and recovery.

Our proposed algorithm utilizes high-resolution 3D point cloud data generated by laser radar sensors to detect and calculate bed angles accurately. This method ensures that even subtle changes in bed elevation are captured with precision, providing healthcare providers with reliable information to make informed decisions about patient positioning. To support the development and validation of our algorithm, we established a comprehensive medical bed angle dataset. This dataset includes a diverse range of bed configurations, covering various angles, baffle positions, and scenarios involving both occupied and unoccupied beds. The richness and diversity of this dataset contribute significantly to the robustness and generalizability of our model. We employed an enhanced version of the A2J (Anchor-to-Joint) network to improve the accuracy of key point estimation. The A2J network was specifically tailored to handle the complexities of point cloud data, enabling more precise localization of bed features and thereby enhancing the overall performance of the bed angle detection system. The bed angle calculation algorithm was optimized for speed and efficiency, achieving a remarkable frame rate of 42 frames per second (FPS) on an NVIDIA GeForce RTX 3080 Ti GPU. This high throughput ensures real-time processing capabilities, making it feasible to deploy the system in dynamic clinical environments where rapid adjustments may be necessary. Experimental results demonstrated that our method achieves an average bed angle detection error of less than 3 degrees, which is well within acceptable limits for medical applications. This level of accuracy is crucial for ensuring proper patient positioning and minimizing the risk of complications such as pressure ulcers and respiratory issues.

The proposed system has been rigorously tested and validated across various simulated and real-world ICU ward environments. These tests confirmed that the method not only achieves higher accuracy on the medical bed angle dataset but also demonstrates practical applicability in actual medical scenarios. By ensuring that bed angles are consistently maintained at optimal levels, the system supports better patient outcomes and reduces the likelihood of secondary complications. Real-time monitoring and adjustment capabilities streamline workflows for healthcare professionals, allowing them to focus more on patient care and less on manual adjustments. The system’s modular design allows for easy integration into existing hospital infrastructure, making it a versatile solution suitable for a wide range of medical settings.

## Figures and Tables

**Table 1:** Comparison of results of different methods on bed angle dataset.

Degree	Towards [13]	Integral [14]	V2V [15]	GAST	A2J [16]	Ours
10°	4.03°	3.65°	2.98°	2.42°	2.37°	2.63°
15°	2.37°	2.43°	1.42°	1.36°	1.19°	1.27°
20°	2.81°	3.01°	1.59°	1.75°	2.01°	1.30°
25°	3.04°	3.74°	3.07°	3.13°	3.19°	2.94°
30°	3.29°	3.95°	2.99°	2.86°	3.01°	2.75°
35°	4.37°	4.63°	3.38°	3.39°	3.26°	3.63°
40°	4.81°	5.12°	4.19°	4.32°	4.24°	3.81°
45°	5.19°	5.32°	4.20°	4.97°	4.32°	4.18°
Total	3.73°	3.98°	2.97°	3.02°	2.94°	2.81°
Time	27 ms	31 ms	68 ms	56 ms	32 ms	39 ms

**Table 2:** Comparison of results of different bed angle and Bed barrier.

Bed Angle		15	20	25	30	35	40	45
Bed barrier Up	Result	13.8	17.6	23.6	28.5	33.9	38.2	41.4
	Error	1.1	2.3	1.3	1.4	1.0	1.7	3.5
Bed barrier Down	Result	12.7	17.3	26.1	31.1	33.6	43.3	40.2
	Error	2.2	2.6	0.8	1.1	1.5	3.3	4.8

## Data Availability

Data are contained within the article.
